# Risk of Flood-Related Diseases of Eyes, Skin and Gastrointestinal Tract in Taiwan: A Retrospective Cohort Study

**DOI:** 10.1371/journal.pone.0155166

**Published:** 2016-05-12

**Authors:** Ling-Ya Huang, Yu-Chun Wang, Chin-Ching Wu, Yi-Chun Chen, Yu-Li Huang

**Affiliations:** 1 Institute of Epidemiology and Preventive Medicine, National Taiwan University, Taipei, Taiwan; 2 Department of Bioenvironmental Engineering, Chung Yuan Christian University College of Engineering, Chung Li, Taiwan; 3 Department of Public Health, China Medical University College of Public Health, Taichung, Taiwan; 4 Department of Health Management, I-Shou University, Kaohsiung, Taiwan; 5 Bachelor’s Degree Program for Indigenous Peoples in Long-term Care, I-Shou University, Kaohsiung, Taiwan; 6 Department of Safety, Health and Environmental Engineering, National Kaohsiung First University of Science and Technology, Kaohsiung, Taiwan; Columbia University, UNITED STATES

## Abstract

Floods are known to cause serious environmental damage and health impacts. Studies on flood-related diseases have been primarily on individual events, and limited evidence could be drawn on potential health impacts from floods using large population data. This study used reimbursement records of one million people of the Taiwan National Health Insurance program to compare incident diseases of the eyes, skin and gastrointestinal (GI) tract associated with floods. Incidence rates for the selected diseases were calculated according to outpatient/emergency visit data. The incidence rates were evaluated by flood status: in 10 days before floods, during floods and within 10 days after the floods receded. Outpatient/emergency visit rates for the eye, skin and GI tract diseases were highest after floods and lowest during floods. Results from multivariate Poisson regression analyses showed that, when compared with the incidence in 10 days before floods, the incidence rate ratios (IRR) of diseases within 10 days after floods were 1.15 (95% confidence interval (CI) = 1.10–1.20) for eyes, 1.08 (95% C.I. = 1.05–1.10) for skin, and 1.11 (95% CI = 1.08–1.14) for GI tract, after controlling for covariates. All risks increased with ambient temperature. V-shaped trends were found between age and eye diseases, and between age and GI tract diseases. In contrast, the risk of skin diseases increased with age. In conclusion, more diseases of eyes, skin and GI tract could be diagnosed after the flood.

## Introduction

Floods may lead to severe changes in environmental sanitation and personal health. A flood may spread contaminants out over the flooded area, deteriorate the drinking water supply and living conditions. Environmental damages and overwhelming effects of floods on infrastructure, overcrowded shelters, poor nutrition and poor sanitary conditions may also increase the risk of infectious diseases [1–30].

Studies found that clinc visits for cellulitis of lower limbs increased after a severe flood and emergency services for gastrointestinal (GI) tract diseases also increased during and after a flood [10–12]. The contaminated water may cause focal retinal whitening and necrotizing retinochoroiditis associated with the *Toxoplasma gondii* infection [13]. The trachoma infection associated with water, sanitation and hygiene may cause a large amount of disability-adjusted life years [14]. Fungal skin infection, bacterial skin infection, eczema, urticaria and scabies have been associated with floods and hurricanes [15,16]. Increased GI diseases, such as enterovirus infection and bacillary dysentery, also have been associated with the flood and heavy precipitation [17,18]. The risk of infections for people in the flood could be especially severe in low income countries [19]. For example, in 2004, an outbreak of cholera and E. coli infection after a flood in Bangladesh caused more than 17,000 cases of diarrhea [20]. In 2010, 2377 patients with scabies were reported in 4 flood-affected regions of Baluchistan in Pakistan [21].

Ambient temperature is an important factor associated with microbial growth, leading to elevated medical utilizations [22–24]. Risks of eye, skin and GI tract infections are increased when average daily temperature is above 30°C [22]. Daily hospital admissions among children also increase with the elevation of ambient temperature [23].

The elderly and children are more vulnerable than other age groups in flood events. The elderly and children are at a higher risk of GI tract infection when the turbidity of water is increased [25,26]. The risk of infections among children playing in the flooded area is elevated [27].

Taiwan is located in the sub-tropic region on the west side of Pacific Ocean with the annual average ambient temperature above 20°C. The Taiwan island receives around 3,000 mm rainfall annually. Most of the rainfalls precipate between April and September. Torrential rainfall and typhoon may lead to local flooding, which occasionally caused severe environmental damages. Increased infections of Leptospirosis and Melioidosis have been reported in Taiwan in severely flooded areas after typhoon [28,29]. Nonetheless, in-depth analyses on potential health impacts relating to flooding using population data have been limited.

This study compared risks of flood-related diseases of eyes, skin and GI tract using a population-based database. In addition, potential effects of demographic characteristics and ambient temperature on flood-related diseases were also explored.

## Materials and Methods

### Health Insurance data

In 1995, the National Health Insurance (NHI) program was initiated in Taiwan, and the program has since covered more than 99% of the 23 million Taiwanese populations [30]. Medical claims records were maintained by National Health Research Institutes (NHRI). We obtained from NHRI the claims data of one million insured people randomly sampled from all people for the period from 1998–2008 in this study. This cohort represented 4.35% of whole insured population with similar age and gender distributions [31]. The medical service records included demographic data of insured people, such as sex, birthdate and residential area, and dates and types of medical service used, diagnoses, dates of admission and discharge for inpatient treatments. The NHRI has scrambled the identification numbers of insured people, changed them into surrogate numbers to protect the privacy of insured people. Therefore, the informed consents of study subjects and ethnic review were exempted. This study was conducted with the approval of the Research Ethics Committee, China Medical University and Hospital (CMU-REC-101-012). We used International Classification of Diseases 9th Revision, Clinical Modification (ICD-9 CM) to screen for the primary diagnosis of diseases of the eyes, skin and GI tract (S1 Table).

### Environmental data

Information of floods was obtained from the Central Weather Bureau (CWB), which administered 8 weather related centers, 25 weather stations, 4 weather radar stations and 214 precipitation monitor sites to document daily 24-hour ambient temperature, relative humidity, rainfall and events such as typhoons. Information of flood events and typhoons was also available from the Ministry of Interior Affairs of Taiwan and news from udndata [32]. For analysis purpose, typhoon events were identified from CWB data. A flood was defined when a storm caused water to flow over normally dry areas. Date of flood onset and receding for the flood-affected counties/cities were identified [22].

### Data analysis

Data analysis first counted the number of floods occurred in all 22 counties and cities and average duration (days) of floods by month in the Taiwan island from 1998–2008. The flood frequency was plotted by county and city. We then plotted the monthly average ambient temperature, rainfall and incidence rates of the diseases of eyes, skin and GI tract to illustrated the general relationships among these factors.

Based on the insurance claims data, we estimated population size in each flood-affected county or city. The population size multiplied by 10 days before the flood, the duration of flood (days), 10 days after floods receded, as the exposure person-days used as the denominator for calculating disease incidence by the flood status. Patients received outpatient/emergency cares for eyes, skin and GI tract diseases were identified for each flood-affected county/city with flood in 10 days before the floods, during the flood periods and in 10 days after the floods. The post flood incidence rate calculated within 10 days after the receding of floods was because the incubation periods of these flood-related diseases could be delayed for several days [17,22]. Incidence rates were calculated in per 100,000 person-days by pooling all these 240 floods and compared among rates 10 days before the floods, during the floods and 10 days after the flood receding using one-way ANOVA.

Multivariable Poisson regression analysis was used to model count variables and determine incidence rate ratio (IRR) and 95% confidence intervals (CI) of diseases by flood status (in 10 days before floods, during floods and within 10 days after floods were receded), daily average temperature (<15, 15–19, 20–24, 25–29, ≥30°C), gender (male/female) and age (<15, 15–64 and ≥65 years).We also included calendar year, month, weekend/holiday, typhoon periods, daily relative humidity, urbanization, gross domestic product (GDP) index and education index as additional controlling factors in the multivariable analysis. For comparisons, the incidence rate of 10 days before the floods was used as the reference (RR = 1). Other reference groups in the model included average daily temperature of 20–24°C, male, and age of 15–64 years. The data of daily average temperature, daily relative humidity and typhoon periods from CWB were used. Age was calculated using the birth date registered in the claims data from NHRI. The information on urbanization level of area with flood was provided by Directorate-General of Budget, Accounting and Statistics (DGBAS), Taiwan [33]. We also used the DGBAS data to calculate annual GDP index in national level and education index in county/city level adapting the method of human development index proposed by the United Nations Development Programme [34].

However, some diseases are associated with seasonality in addition to the flood association. Data analysis further demonstrated the seasonality of diseases by calculating the monthly mean diseases of the eyes, skin and GI from 1998–2008. All statistical analyses were performed using SAS version 9.1 (SAS Institute Inc., Cary, NC, USA), and the value of α was taken as 0.05.

## Results

**Fig 1:**
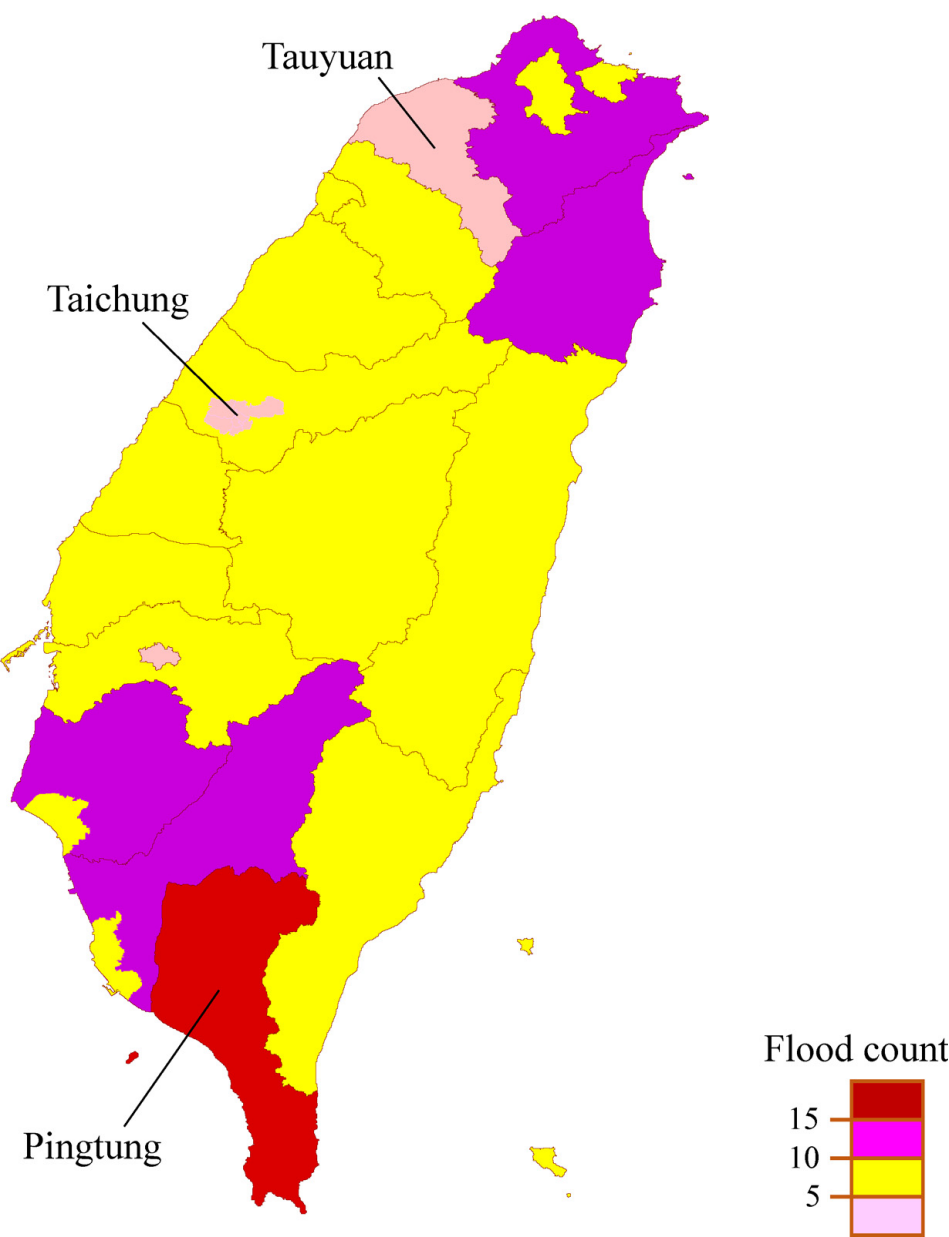
The frequencies of flood by county and city in Taiwan Island during 1998 to 2008.

There were 240 floods occurred from 1998 to 2008 in Taiwan, with the average flooding period of 3.9 ± 3.3 days, mainly in summers and autumns peaked in August (Table 1). Fig 1 shows the distribution of these floods by county/city, ranging from <5 floods in Tauyuan county in the northwest and Taichung city in the central area to >15 floods in Pingtung of southwest area.

**Table 1:** Mean durations of floods in Taiwan estimated by month and frequency during 1998–2008.

	Flood	
		Duration time, day
Month	number	Mean (SD)
1	0	-
2	13	1.0 (0)
3	0	-
4	0	-
5	5	3.6 (0.6)
6	46	7.8 (4.9)
7	55	3.2 (1.6)
8	64	2.1 (1.5)
9	25	3.4 (2.8)
10	32	4.2 (1.3)
11	0	-
12	0	-
Total	240	3.9 (3.3)

**Fig 2:**
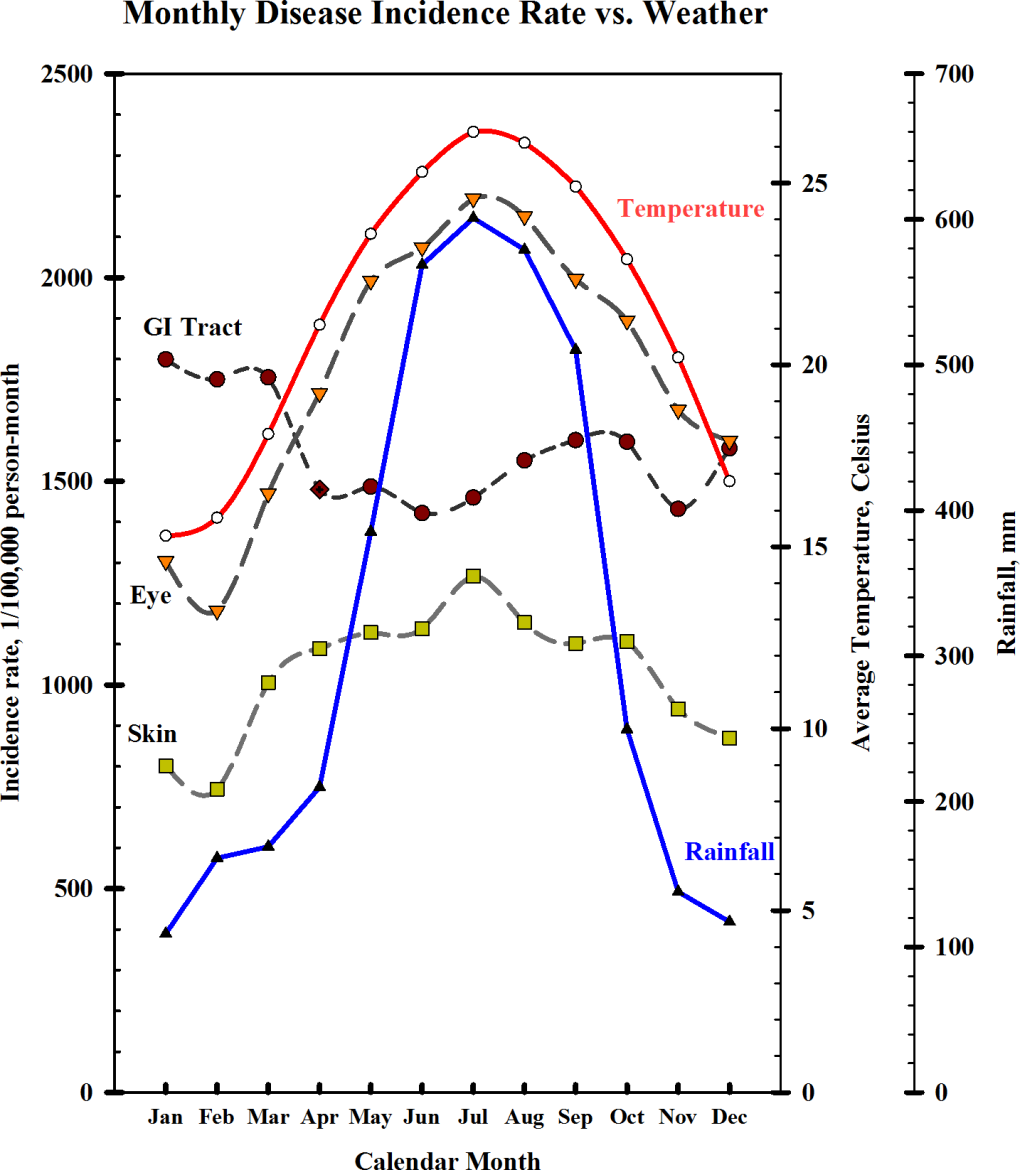
Monthly average incidence rates of eye, skin and GI diseases associated with monthly weather status from 1998 to 2008 in Taiwan.

Fig 2 shows monthly average incidence rates of diseases for eyes, skin and GI tract associated with monthly average temperatures and rainfalls in Taiwan. The monthly average temperatures ranged from 15.3°C (SD = 2.3°C) in January to 26.4°C (SD = 2.4°C) in July. The monthly rainfall was also the lowest in January (average 109, SD 106 mm) and the highest in July (average 601, SD 532 mm). The monthly incidence rates of the eyes and skin were associated with temperatures and rainfalls. On the other hand the rates for the GI disease were greater in cold months, particularly in January and February.

Table 2 presents incidence rates of skin, eyes and GI tract diseases for patients receiving care at outpatient clinics and emergency room in 10 days before the floods, during floods and within 10 days after the floods receded. Based on the 4.35% subsample of the population, we found that the unadjusted incidence rates of the 3 types of diseases among 3 measured periods were all lower during the flood than both before and after the floods. The differences among the 3 periods were significant for skin and GI tract infections (*P =* 0.006 and 0.01, respectively), but not for eye infections (*P =* 0.25).

**Table 2:** The unadjusted incidence rates of outpatient/emergency visits for eyes, skin and gastrointestinal tract diseases by flood status based on the subsample of the population.

Disease	Incidence rate (per 100,000 person-days)			
	Mean (standard deviation)			
	10 days before flood^a^	During flood	Within 10 days After flood	p-value^b^
**Eye**	41.83 (40.54)	34.90 (37.99)	42.45 (40.93)	0.25
**Skin**	78.10 (36.97)	65.06 (39.09)*	78.86 (36.98)	0.006
**GI tract**^c^	56.91 (23.58)	49.65 (24.74)*	58.07 (22.53)	0.01

^a^10 days before floods excluded the days during typhoons and 10 days after typhoons

^b^ by one-way ANOVA

^c^GI: gastrointestinal

* The incidence rate during floods was different compared with 10 days before floods and within 10 days after floods.

Table 3 shows the multivariable Poisson regression analysis estimated IRRs of diseases by flood status, temperature, sex, and age. Compared to the period of 10 days before floods, risks of the three study disorders were lower during floods, significant for diseases of eyes (IRR = 0.90, 95% CI = 0.85–0.95) and skin (IRR = 0.92, 95% CI = 0.90–0.95). The IRRs of the three diseases post floods were all significantly greater than the non-flood period: 1.15 (95% CI = 1.10–1.20) for eyes, 1.08 (95% CI = 1.05–1.10) for the skin and 1.11 (95% CI = 1.08–1.14) for the GI tract. Risks of all three diseases increased with temperature. Relative to males, females were at higher risk for eyes and GI diseases, but at lower risk for the skin disease. Young children and the elderly were at higher risk for eye and GI infections than residents aged 15–64 years, while the risk of the skin disease increased with age.

**Table 3:** Multivariable Poisson regression analysis estimated incidence rate ratio of infections by flood status, temperature, sex and age controlling for other covariates^a^.

Risk factors	IRR (95% CI)^b^		
	Eyes	Skin	GI^c^ tract
**Flood status**			
During flood	0.90 (0.85–0.95)	0.92 (0.90–0.95)	0.98 (0.95–1.01)
10 days after flood	1.15 (1.10–1.20)	1.08 (1.05–1.10)	1.11 (1.08–1.14)
10 days before flood	1.00	1.00	1.00
**Average daily temperature, °C**			
< 15	0.58 (0.47–0.71)	0.71 (0.64–0.79)	0.98 (0.91–1.05)
15–19	0.58 (0.53–0.64)	0.70 (0.66–0.73)	0.96 (0.91–1.004)
20–24	1.0	1.0	1.0
25–29	1.09 (1.05–1.13)	1.09 (1.06–1.11)	1.03 (0.99–1.05)
≥ 30	1.36 (1.16–1.58)	1.14 (1.05–1.23)	1.24 (1.15–1.34)
**Demographic factors**			
**Gender**			
Male	1.0	1.0	1.0
Female	1.37 (1.33–1.41)	0.90 (0.88–0.92)	1.11 (1.09–1.14)
**Age, years**			
< 15	1.75 (1.69–1.81)	0.73 (0.71–0.75)	1.90 (1.86–1.95)
15–64	1.0	1.0	1.0
≥ 65	2.61 (2.51–2.73)	1.57 (1.53–1.62)	1.53 (1.48–1.58)

^a^adjusted by calendar year, month, weekend/holiday, typhoon periods, daily relative humidity, urbanization, GDP index and education index.

^b^IRR: incidence rate ratio; CI: confidence interval.

^c^GI: gastrointestinal.

## Discussion

This population-based study used medical insurance claims data to explore potential associations between floods and diseases of the skin, eyes and GI tract. The use of medical insurance claims records reduced potential self-reporting bias, and the large database allowed the exploration of diseases that may be associated with floods.

From 1998 to 2008, the south western Taiwan was the major flood-affected area among all counties and cities ever flooded in the Taiwan island. Most floods in Taiwan occur during the summer months of June, July and August, which is the period with the highest ambient temperature and rainfall events, conditions that favor infections.

Increased medical service utilizations were found to be associated with floods, with lower incident diseases of the eye, skin and GI tract during floods, but higher within 10 days after floods. Inconvenience to seek care during flood and the incubation period of diseases may lead to lower incidences of selected diseases during floods.

Floods may wash contaminants into the living environment, discharge sewage and introduce pathogens to surface waters and other materials. In addition, the flood may damage water treatment systems and contaminate drinking water [35]. Residents are likely to contact with polluted water but lack fresh water to drink. Various studies have reported associations between contaminated water caused by heavy rainfalls or floods and increased risks of waterborne diseases [1,10,11,16,17,35–38]. The diseases of eyes, skin and GI are likely results from exposure to contaminated water and materials during the flood and in the post-flood cleanup process. Huang et al. reported that medical services of conjunctivitis, trachoma, and other inflammation of the eyelids increased during and after water outage [22].

Conjunctivitis and trachoma increase for people lack of fresh water to clean-up [39].The estimated worldwide disability-adjusted life year of trachoma associated with water, sanitation and hygiene was 1,239,000 years in 1999 [14]. In Brazil, the increased cases of focal retinal whitening and necrotizing retinochoroiditis identified in a flood have been associated with the *Toxoplasma gondii* infection in the contaminated water [13]. A study found patients with cellulitis of lower limbs after a severe flood 6.2-fold more likely to have immersed the limbs in the flood water than non-patients [3]. Fungal skin infection, bacterial skin infection, eczema, urticarial and scabies also have been associated with flood or hurricane [15,16]. In Denmark, GI tract diseases increased in people who contacted flood water but failed to wash their hands [12]. Wade et al. reported that emergency services for gastrointestinal (GI) tract diseases increased during flood period and within 4 days after the flood [10,11].

In this study, the incidence rate of GI tract diseases is higher in January, February and March than in other months (Fig 2). There was no flood in January and March. In Taiwan, the norovirus and rotavirus epidemics occur mainly from November to March and peak in January [40]. People consume more foods which may associate with GI tract symptoms during the holidays of lunar year festivals (usually in January or February).

Warm ambient temperature is known to encourage microbial growth. In this study, risks of the eye, skin and GI tract diseases significantly increased when average daily temperature reached above 30°C. In a Peruvian study, the relative risk of daily hospital admissions among children ranged from 1.04 to 1.12 when ambient temperature increased by 1°C [23]. Hashizume et al. [24] have also reported a positive association in daily cases of non-cholera diarrhea with average ambient temperature in Bangladesh.

Our data show that the incidence rates of the study diseases were different between males and females. Women had overall higher rates of the eye and GI tract diseases; men had an overall higher rates of skin diseases than males. Among all age groups, the elderly were most susceptible to all three types of diseases. Children under 15 years old also had higher rates of eye and GI tract diseases, but had a lower rate of skin diseases. However, the real impact of floods on the risk of selected diseases among subgroups needs further study.

Most floods occurring in Taiwan are associated with heavy rainfalls due to typhoons in warmer months. However, the diseases can occur without floods, as Fig 2 shows that the study diseases vary by season and month. This study has considered other potential confounding factors in Poisson regression analysis, including calendar year, month, weekend/holiday, typhoon periods, daily relative humidity, urbanization, GDP index and education index as additional controlling factors in addition to flooding events. We consider month as a more appropriate factor than season.

This study is limited in several aspects. First, the official identified flood events were limited to county/city level and therefore the whole county/city residents were assumed to be exposed to the flood. The association between flood events and the risks of the eye, skin and GI tract diseases might be underestimated due to non-differential exposure misclassification. Second, we estimated post-flood effect by calculating cases diagnosed within 10 days after flood. Cases with the disease with short incubation period may seek care during the flood period if the disorder is severe, while some cases may visit clinic after the flood. Therefore, misclassification of incident cases between flood period and post flood period is possible. Finally, the insurance claims data were used to identify cases of selected diseases, and laboratory tests for case confirmation were not included in the analyses. The causes of diseases were not examined in this study. In some cases, people suffering from diseases may not seek medical assistance, thus the incidence rates may have been underestimated.

## Conclusions

This study examined the risk of diseases with respect to its association with flooding. The results suggest that incident diseases of eyes, skin and GI tract may increase within 10 days after floods receded.

## Supporting Information

S1 Table The summarized ICD-9 CM codes for selected diseases of eye, skin and gastrointestinal tract. (DOCX)
