# Supplementary material for: Risk of Flood-Related Diseases of Eyes, Skin and Gastrointestinal Tract in Taiwan: A Retrospective Cohort Study
Source: PLoS One. 2016 May 12;11(5):e0155166. doi: 10.1371/journal.pone.0155166 (PMC4865035; doi:10.1371/journal.pone.0155166)
Supplement: S1 Table — (DOCX) [file pone.0155166.s001.docx]

**S1 Table The summarized ICD-9 CM codes for selected diseases of eye, skin and gastrointestinal tract**

| **Type of diseases** | **Selected diseases (ICD-9 CM codes)** |
| --- | --- |
| **Eyes** | Trachoma (076), Acute conjunctivitis (3720), Infective dermatitis of eyelid of types resulting in deformity (3734), Other infective dermatitis of eyelid (3735), Parasitic infestation of eyelid (3736) |
| **Skin** | Dermatophytosis (110), Dermatomycosis, other and unspecified (111), Acariasis (133) , Carbuncle and furuncle (680), Cellulitis and abscess of finger and toe (681), Other cellulitis and abscess (682), Acute lymphadenitis (683), Impetigo (684), Pilonidal cyst (685), Other local infections of skin and subcutaneous tissue (686), Rash and other nonspecific skin eruption (7821) |
| **Gastrointestinal tract** | Cholera (001), Typhoid and paratyphoid fevers (002), Other salmonella infections (003), Shigellosis (004), Other food poisoning (bacterial) (005), Amebiasis (006), Other protozoal intestinal diseases (007), Intestinal infections due to other organisms (008), Infectious colitis, enteritis and gastroenteritis (009), Gastritis and duodenitis (535), Persistent vomiting (5362), Regional enteritis (555), Toxic gastroenteritis and colitis (5582), Other and unspecified noninfectious gastroenteritis and colitis (5589), Peritonitis (567), Unspecified disorder of peritoneum (5689), Gastrointestinal hemorrhage (578), Symptoms involving digestive system (787) |
